# INDIGENOUS RESILIENCY AGAINST THE SOCIAL, BEHAVIORAL, AND MENTAL DETERMINANTS OF HEALTH DURING COVID-19 PANDEMIC

**DOI:** 10.1093/geroni/igae098.1828

**Published:** 2024-12-31

**Authors:** Kristi Kaapu, Catherine McKinley, Michelle Johnson-Jennings

**Affiliations:** Tulane University, New Orleans, Louisiana, United States; Tulane University, New Orleans, Louisiana, United States; University of Washington, Seattle, Washington, United States

## Abstract

U.S. Indigenous people have been disproportionately impacted by the COVID-19 pandemic, experiencing higher rates of infection and hospitalization, being vulnerable to mental health symptoms, including psychological stress and emotional health, and experiencing higher rates of loss and death. Structural inequities have contributed to these negative COVID-19 health outcomes; however, consistent with the Framework of Historical Oppression, Resilience, and Transcendence (FHORT), Indigenous people offset these disparities through positive coping, adaptation, and resiliency interwoven between individual, family and community ecological levels. This research assessed interconnections of structural inequality and associated disruptions in relation to Indigenous wholistic wellness, primarily around healthcare discrimination, medical mistrust, mental health, and loss and death. Thirty-one head-of-household women participated in community-based, critical ethnographic interviews to assess disparities associated with COVID-19. All participants were in caregiving roles and ranged in age from 29-56 years old. The following themes emerged: (a) negative medical and healthcare experiences; (b) connections between physical and mental health; (c) worry and depression; (d) loss of structure and self; (e) disrupted burial rituals; (f) death, grief, and extensive losses; and (g) resiliency. Results indicate disparities associated with the pandemic emerged in large part from systemic inequity; however, Native American women demonstrated resiliency by advocating for themselves, problem solving, self-coping, and upholding Indigenous values. With lower life expectancy of Indigenous peoples, this study informs both future generations and interventions, promoting traditional and cultural protective factors to offset the impact of historical trauma and oppression, including healing across all ecological domains including physical, mental, emotional, and spiritual aspects.

